# Imaging modality-dependent carotid stenosis severity variations against intravascular ultrasound as a reference: Carotid Artery intravasculaR Ultrasound Study (CARUS)

**DOI:** 10.1007/s10554-023-02875-1

**Published:** 2023-08-21

**Authors:** Lukasz Tekieli, Anna Kablak-Ziembicka, Wladyslaw Dabrowski, Karolina Dzierwa, Zbigniew Moczulski, Malgorzata Urbanczyk-Zawadzka, Adam Mazurek, Justyna Stefaniak, Piotr Paluszek, Maciej Krupinski, Tadeusz Przewlocki, Piotr Pieniazek, Piotr Musialek

**Affiliations:** 1 Department of Cardiac and Vascular Diseases, Institute of Cardiology, Jagiellonian University Medical College, Krakow, Poland; 2 Department of Interventional Cardiology, Institute of Cardiology, Jagiellonian University Medical College, Krakow, Poland; 3 John Paul II Hospital, Krakow, Poland; 4 Noninvasive Cardiovascular Laboratory, John Paul II Hospital, Krakow, Poland; 5 KCRI Angiographic and IVUS Core Laboratory, Krakow, Poland; 6 Department of Radiology and Diagnostic Imaging, John Paul II Hospital, Krakow, Poland; 7 Data Management and Statistical Analysis (DMSA), Krakow, Poland; 8 Department of Bioinformatic and Telemedicine, Jagiellonian University, Krakow, Poland; 9 Department of Vascular Surgery and Endovascular Interventions, John Paul II Hospital, Krakow, Poland

**Keywords:** Carotid stenosis severity, Duplex ultrasound, Computed tomography angiography, Catheter angiography, Intra-arterial angiography, Intravascular ultrasound

## Abstract

**Purpose:**

Different non-invasive and invasive imaging modalities are used to determine carotid artery stenosis severity that remains a principal parameter in clinical decision-making. We compared stenosis degree obtained with different modalities against vascular imaging gold standard, intravascular ultrasound, IVUS.

**Methods:**

300 consecutive patients (age 47–83 years, 192 men, 64% asymptomatic) with carotid artery stenosis of “ ≥ 50%” referred for potential revascularization received as per study protocol (i) duplex ultrasound (DUS), (ii) computed tomography angiography (CTA), (iii) intraarterial quantitative angiography (iQA) and (iv) and (iv) IVUS. Correlation of measurements with IVUS (r), proportion of those concordant (within 10%) and proportion of under/overestimated were calculated along with recipient-operating-characteristics (ROC).

**Results:**

For IVUS area stenosis (AS) and IVUS minimal lumen area (MLA), there was only a moderate correlation with DUS velocities (peak-systolic, PSV; end-diastolic, EDV; r values of 0.42–0.51, *p* < 0.001 for all). CTA systematically underestimated both reference area and MLA (80.4% and 92.3% cases) but CTA error was lesser for AS (proportion concordant-57.4%; CTA under/overestimation-12.5%/30.1%). iQA diameter stenosis (DS) was found concordant with IVUS in 41.1% measurements (iQA under/overestimation 7.9%/51.0%). By univariate model, PSV (ROC area-under-the-curve, AUC, 0.77, cutoff 2.6 m/s), EDV (AUC 0.72, cutoff 0.71 m/s) and CTA-DS (AUC 0.83, cutoff 59.6%) were predictors of ≥ 50% DS by IVUS (*p* < 0.001 for all). Best predictor, however, of ≥ 50% DS by IVUS was stenosis severity evaluation by automated contrast column density measurement on iQA (AUC 0.87, cutoff 68%, *p* < 0.001). Regarding non-invasive techniques, CTA was the only independent diagnostic modality against IVUS on multivariate model (*p* = 0.008).

**Conclusion:**

IVUS validation shows significant imaging modality-dependent variations in carotid stenosis severity determination.

**Supplementary Information:**

The online version contains supplementary material available at 10.1007/s10554-023-02875-1.

## Introduction

Despite growing understanding of the role of plaque morphology [1–10], stenosis severity remains the fundamental factor in clinical decision-making with regard to recommending medical or interventional therapy to symptomatic and asymptomatic patients with internal carotid artery (CA) atherosclerosis [2, 3, 11–13].

In the past, major trials used mostly intraarterial quantitative angiography (iQA) for grading ICA stenosis [14, 15]. However, iQA poses a non-negligible risk of cerebral ischemic complications (typically ≈0.3%) [16–19]. In addition, iQA needs to be obtained in multiple projections to determine the maximal diameter stenosis [20, 21]. Considering safety and economic reasons, iQA has been largely replaced by non-invasive methods including Doppler ultrasound (DUS), computed tomography angiography (CTA) and magnetic resonance angiography (MRA) [22]. These modalities have been employed in more recent trials; often different ones in different clinical trials [14, 15, 23–26]. Even more importantly, the non-invasive modalities are routinely used these days for clinical decision-making including decisions on carotid revascularization in primary and secondary stroke prevention [2, 3, 11–13].

It has been clear for some time now that discrepancies may exist between the different techniques of carotid stenosis severity determination [22, 27–29]; this issue, however, has not been systematically investigated.

## Materials and methods

### Design

**Fig. 1:**
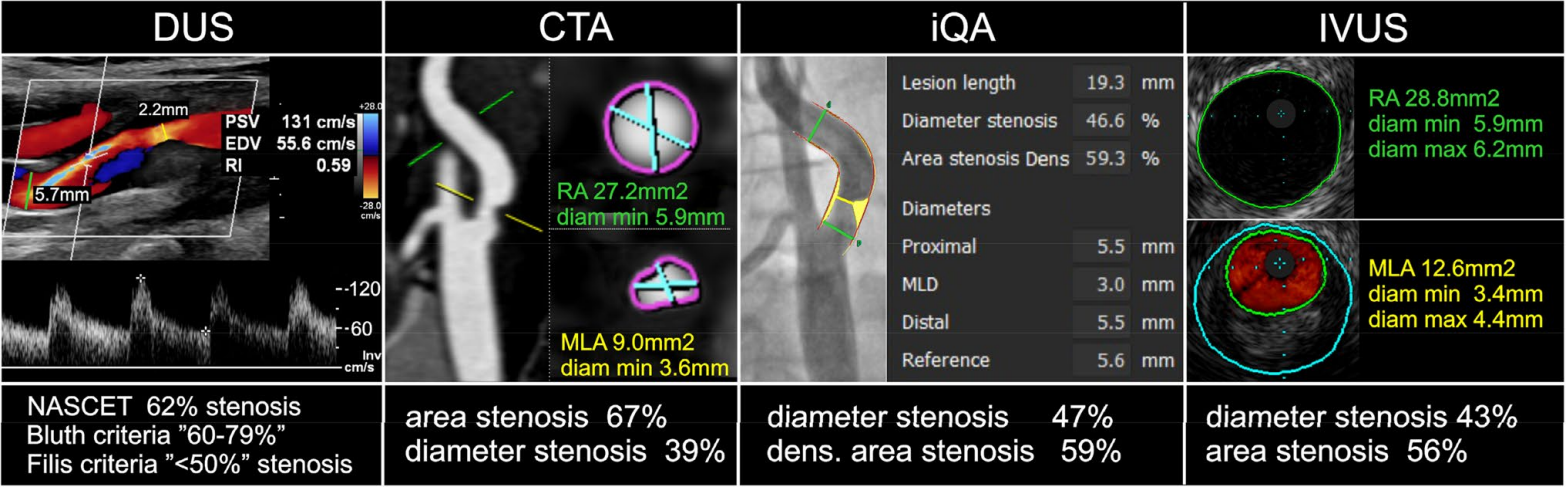
Multimodality imaging in a 68-year-old asymptomatic man referred for potential carotid revascularization and enrolled in the CARUS study. DUS examination shows a peak systolic velocity (PSV) of 1.31 m/s and peak end-diastolic velocity (EDV) of 0.56 m/s; DUS-determined diameter stenosis is 62% according to NASCET [12]. With these values, the patient could be enrolled (or not enrolled) into major DUS-based trials (that used local-lab criteria), grounding today’s clinical guidelines [2, 3, 11–13]. The inclusion to/exclusion from, for instance ACST-1 [23] or ACST-2 [25], would depend on what particular criteria are locally applied, as the stenosis is 60–79% according to Bluth [31], 50–69% according to Grant [32] or Oates [33] but < 50% by Filis [34]. CTA, that is recommended in particular in cases of “uncertainty” in basing clinical decision on DUS [2, 3, 11–13], shows area stenosis (a typical CTA report parameter) of 67%, indicating a “significant” lesion. However, CTA determination of diameter stenosis (ie., the fundamental stenosis severity parameter used in the early trials of carotid revascularization [14, 23]) reveals the value of “39%” (a “non-significant” stenosis). The relationship between diameter stenosis and area stenosis is known to be fundamentally determined by π(d/2)^2^ (leading to different numeric “values” of stenosis severity dependent on the calculation methods [35, 36]; however, the guidelines do not precise which of the 2 stenosis severity parameters by CTA should be used for clinical decision-making [2, 3, 11–13, 37]. Quantitative intra-arterial angiography (used in some major studies such as CREST to resolve discrepancies between non-invasive stenosis severity determination techniques [26]) shows, in the angiographic projection capturing the greatest stenosis severity, a “non-significant” lesion of “47%” diameter stenosis. However, the iQA stenosis severity reading, based on contrast column density (that might offer a higher precision than conventional 2D-based parameters), demonstrates stenosis of “59%” that would already qualify this patient for intervention (had they been symptomatic). IVUS verification shows diameter stenosis of 43% and area stenosis of 56%, with a stable (low-risk) fibroatheroma (see Suppl Fig. 1) on “virtual histology” modality [38]. Note differences between the different techniques in establishing parameters such as reference diameter (area) and minimal lumen diameter (area) that contribute to the discrepancies. The patient was not subjected to any type of intervention; he recieved maximized medical therapy and he is followed up yearly with duplex ultrasound in outpatient clinic. Clinical symptoms or lesion progression would likely trigger intervention. For overall CARUS study data see Fig. 2–6 and Suppl Fig. 2–6. See text for details

This was a monocentric prospective study enrolling consecutive neurologically asymptomatic or symptomatic patients with CA referral stenosis of at least “50%” in the context of potential revascularization. A systematic evaluation was performed of the differences in stenosis severity determination (clinically-relevant example in Fig. 1) using routine imaging modalities—duplex ultrasound (DUS), computed tomography angiography (CTA), and intra-arterial quantitative angiography (iQA)—against the vascular imaging gold standard, intravascular ultrasound (IVUS) [28–30]. Target population of 300 participants was recruited over the period of 19 months.

**Fig. 2:**
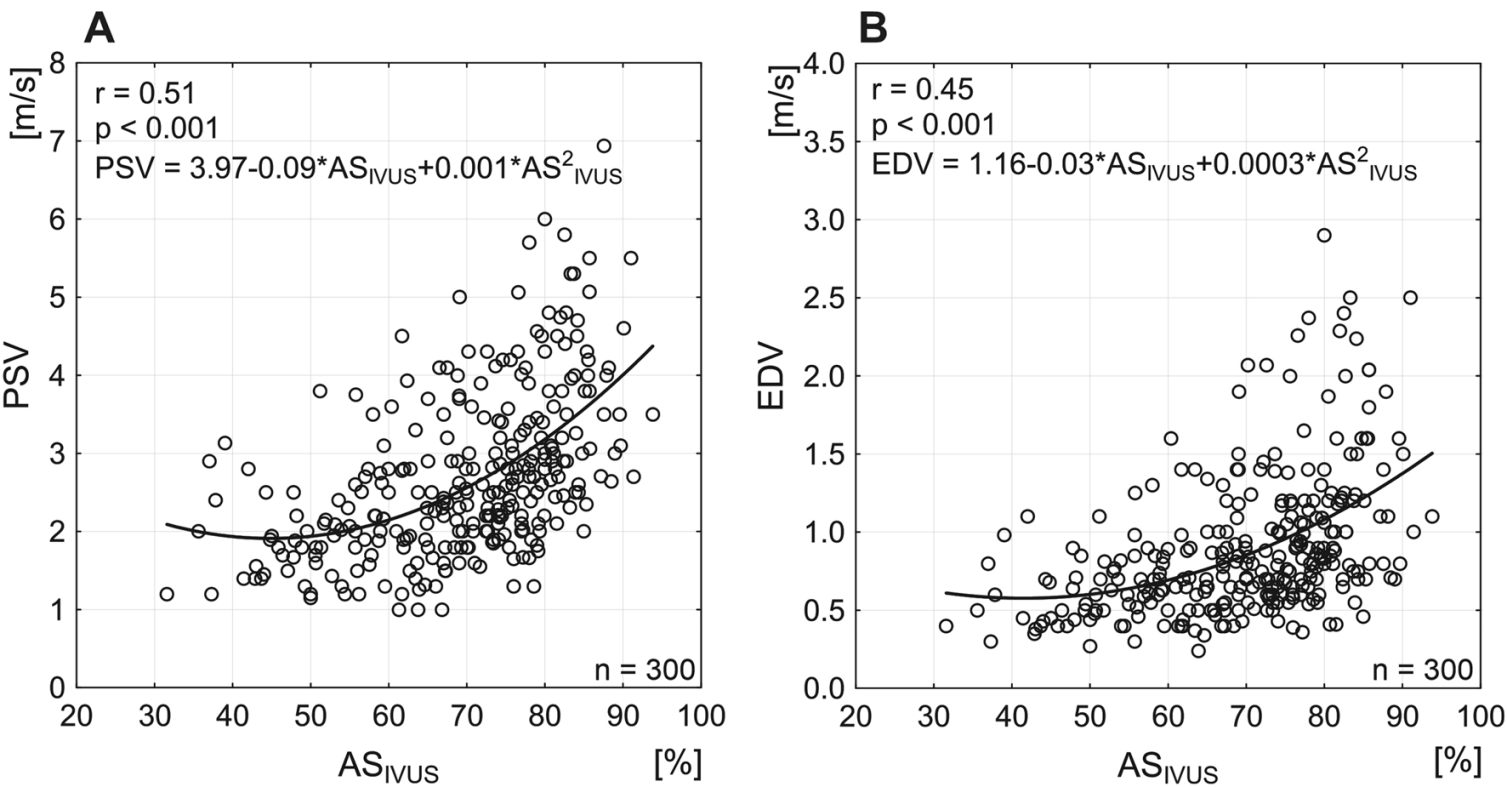
Relation between DUS flow velocities (PSV, EDV) and area stenosis measured by IVUS. Note that overall correlations here are moderate, curvilinear and highly statistically significant for both comparison (correlation coefficient “r” 0 indicates absence of any correlation whereas 1 indicates a perfect correlation). Best-fit mathematical formulas are provided for both relationship

Patients with highly calcified lesions [40] that precluded reliable assessment of velocities as per agreement of 2 experienced ultrasonographers, and those with chronic kidney disease with glomerular filtration rate < 30 ml/minute, were excluded.

309 consecutive patients meeting clinical and non-invasive imaging inclusion/exclusion criteria were enrolled to obtain 300 data-sets suitable for analysis. DUS, CTA, and subsequently iQA and IVUS were performed (all examinations within 1 month). In 8 subjects, iQA revealed an ‘artery near-occlusion’ precluding IVUS visualization of the native carotid lesion. In those cases, IVUS visualization would have required, by agreement of 2 experienced operators, lesion predilatation with a small-diameter balloon (thus losing any relationship between the basal non-invasive measurements). In 1 other patient, there was a failure to save IVUS data. Thus 9 initially considered patients (2.9%) were screening failures in the context of obtaining required data-sets. In the case of bilateral disease, the more severe lesion was evaluated unless this was considered too tight for a safe passage with the IVUS transducer. Medical treatment prior to iQA and IVUS involved aspirin and a statin. The majority of patients (262/300, 87.3%) were also on an angiotensin-converting enzyme inhibitor or angiotensin receptor blocker.

The study was accepted by the institutional Ethics Committee and all participants provided written informed consent to participate. This work was supported by the National Committee for Scientific Research (PL-N402-184234), the Polish Cardiac Society/Servier Clinical Research in Atherosclerosis grant (to PM), the National Science Centre (2022/06/X/NZ5/00583) and Jagiellonian University Medical College (K/ZDS/007819). The authors have no relevant financial or non-financial interests to disclose.

### DUS examination

DUS scanning was performed with a Toshiba Aplio PowerVision ultrasound machine (Toshiba Medical Systems Co., Ltd., Tokyo, Japan) equipped with a 4–11 MHz linear-array transducer in a certified vascular ultrasound imaging lab by one of two experienced operators working together on a daily basis. In case of doubt, the other DUS operator was consulted and an agreement was reached. The Doppler waveform was obtained with an angle of insonation equal to 60°, angles between 45° and 60° were considered acceptable in case of anatomic constraints [32, 37]. The CA was sampled through the region of stenosis completely until the distal end of the plaque is visualized, to ensure that the site of highest velocity has been located. The highest flow velocities – end diastolic velocity (EDV) and peak systolic velocity (PSV) were recorded. In the first 100 study arteries considered by agreement of the 2 DUS operators appropriate for optimal measurement of diameter stenosis according to the NASCET method, DUS minimal lumen diameter (MLD) and DUS distal reference diameter (RD) were determined by agreement of the ultrasonographers.

### CTA

Image acquisition of the supra-aortic vessels was obtained with a 64-multi-detector-row CT system (Somatom 64, Siemens, Erlangen, Germany) using a routine imaging protocol. CTA measurements (including reference area, RA; minimal lumen area, MLA and area stenosis AS) were performed by agreement of 2 senior radiologists with > 20 years and > 15 years of experience in reporting carotid CTA.

### iQA

After obtaining transfemoral or transradial access, unfractionated heparin (UFH) was routinely administered at the dose of 5000 IU. In case of cerebral protection device use or post-imaging proceeding to intervention, UFH dose was further titrated to achieve an activated clotting time of at least 250 s. Selective digital angiography of the index carotid artery was performed using Coroscop or Axiom Artis Zee angiograph (Siemens) in multiple (median 4) angulated projections to define the narrowest lumen diameter while minimizing foreshortening and avoiding an overlap of side branches. The view where the stenosis was tightest [18, 19] was used for quantitative measurements (Quantcor QA v5.0, Siemens). Measurements (including RD, MLD, DS, RA, MLA, AS) were performed offline by agreement of two Angiographic Core Lab analysts and were then verified by an angiographic corelab supervisor.

### IVUS

Details regarding IVUS images acquisition and analysis are provided in Ref. [39]. Consistent with our prior experience [41], the decision to use a neuroprotection device for IVUS imaging was based on the lesion morphology, severity and the presence/absence of a history of ipsilateral clinical symptoms or asymptomatic cerebral infarct, and it was left to the operator performing the case.

In brief, a commercially-available rapid-exchange IVUS catheter (3.5F, scanner diameter 1.15 mm Eagle Eye Gold or Platinium, Volcano-Philips Corp.) was introduced to the index ICA over a 0.014-inch coronary guidewire (in case of unprotected imaging or imaging under proximal cerebral protection) or, in case of distal embolic protection device use, over the wire of the protective filter. At least two IVUS runs with automatic motorized pullback were performed with the speed of 0.5 mm/sec. ChromaFlo application (Volcano-Philips) was routinely used in one IVUS run to improve the determination of the interface between the lumen and the vessel wall or atherosclerotic plaque [39, 42, 43]. In addition, in short, or ambiguous lesions, a very slow manual pullback was additionally performed in order not to miss the minimal lumen site [40]. IVUS measurements of the minimal lumen area (MLA) and distal reference area (RA) were performed at maximal vessel diastole using QIvus software (v.2.0, Medis Medical Imaging Systems). IVUS measurements were performed by agreement of two IVUS corelab analysts with > 10 years of experience in carotid IVUS analysis and were further approved by the IVUS corelab supervisor. The analysts performing DUS, CTA, iQA and IVUS measurements were blinded against one another.

The % diameter stenosis (DS) was computed as [(RD-MLD)/RD]*100% according to the North American Symptomatic Carotid Endarterectomy Trial (NASCET) method [14]. The % area stenosis (AS) was computed as [1 − (MLA/RA)]*100%.

### Data display and statistical analysis

Categorical variables were presented as numbers and percentages. Continuous variables were expressed, unless specified otherwise, as median and quartiles (Q1–Q3). Receiver operating characteristic (ROC) curves were constructed to assess the accuracy of DUS, CTA and iQA measurements in comparison to IVUS. The overall accuracy was expressed by the area under the ROC curve (AUC; ranging from 0.5 [no relationship] to 1.0 [perfect relationship]). The correlation between DUS/CTA and IVUS was presented as a correlation coefficient (‘r’; ranging from 0 [no correlation] to 1.0 [perfect correlation]). Furthermore, the agreement between quantitative measurements was displayed using histograms and Bland–Altman plots [44]. In addition, the proportion of measurements concordant with IVUS along with those under/overestimated was calculated and displayed in a bar graph format. Concordance with IVUS measurement was defined as a value within ± 10% of the IVUS measurement; values falling below were considered underestimated whereas those falling above were considered overestimated against IVUS as a reference. Mann–Whitney U test was used to assess differences in the distributions between the two groups. A univariate and multivariable model was used to evaluate the predictive value of DUS and CTA for ≥ 50% and ≥ 75% AS by IVUS. The thresholds of 75% AS and 50% DS were used because (with area calculated as π (D/2)) 75% area stenosis corresponds to 50% diameter stenosis [35, 36].

## Results

A complete imaging package, including DUS PSV and EDV, CTA, iQA and IVUS was obtained in 300 patients; whose clinical characteristics are given in Table 1.

**Table 1:** Clinical characteristics of the CARUS study patients

n		300
Age, years, median	[Q1–Q3]	66 [60.0–72.0]
Women	n (%)	108 (36.0)
Symptomatic	n (%)	108 (36.0)
Arterial hypertension	n (%)	266 (88.7)
Diabetes	n (%)	96 (32.0)
on insulin	n (%)	31 (10.3)
CAD		201 (67.0)
h/o myocardial infarction	n (%)	76 (25.3)
smoking (current or past)	n (%)	160 (53.3)
PAD	n (%)	45 (15.0)
BMI, median	[Q1–Q3]	27.7 [25.7–30.1]
Creatinine, μmol/L, median	[Q1–Q3]	85 [74–101]
30 ≤ eGFR < 60, mL/min	n (%)	65 (22.2)

CAD, coronary artery disease; PAD, peripheral artery disease; BMI, body mass index; eGFR, estimated glomerular filtration rate

Table 2 shows baseline characteristics of the study lesions. iQA imaging (that routinely involved anticoagulation with ≥ 5000 IU UFH) and IVUS imaging (with elective use of a proximal or distal cerebral protection device) were uncomplicated. 112 (37.3%) study lesions were evaluated with IVUS in absence of a cerebral protection device use. IVUS imaging was filter-protected in 142 (47.3%) cases, whereas proximal protection was used in 46 (15.3%) cases.

**Table 2:** Baseline characteristics of study lesions

n		300
RICA, n, %		137 (45.7)
LICA, n, %		163 (54.3)
PSV, m/s, median	[Q1–Q3]	2.5 [1.9–3.3]
EDV, m/s, median	[Q1–Q3]	0.9 [0.6–1.2]
DUS diameter stenosis, % (NASCET), median	[Q1–Q3]	69.9 [60.5–77.1]
CTA area stenosis (%), median	[Q1–Q3]	73 [63–81]

PSV, Peak Systolic Velocity; EDV, End-Diastolic Velocity; DUS, Duplex Ultrasound; NASCET, North American Symptomatic Carotid Endarterectomy Trial method [14]

**Fig. 3:**
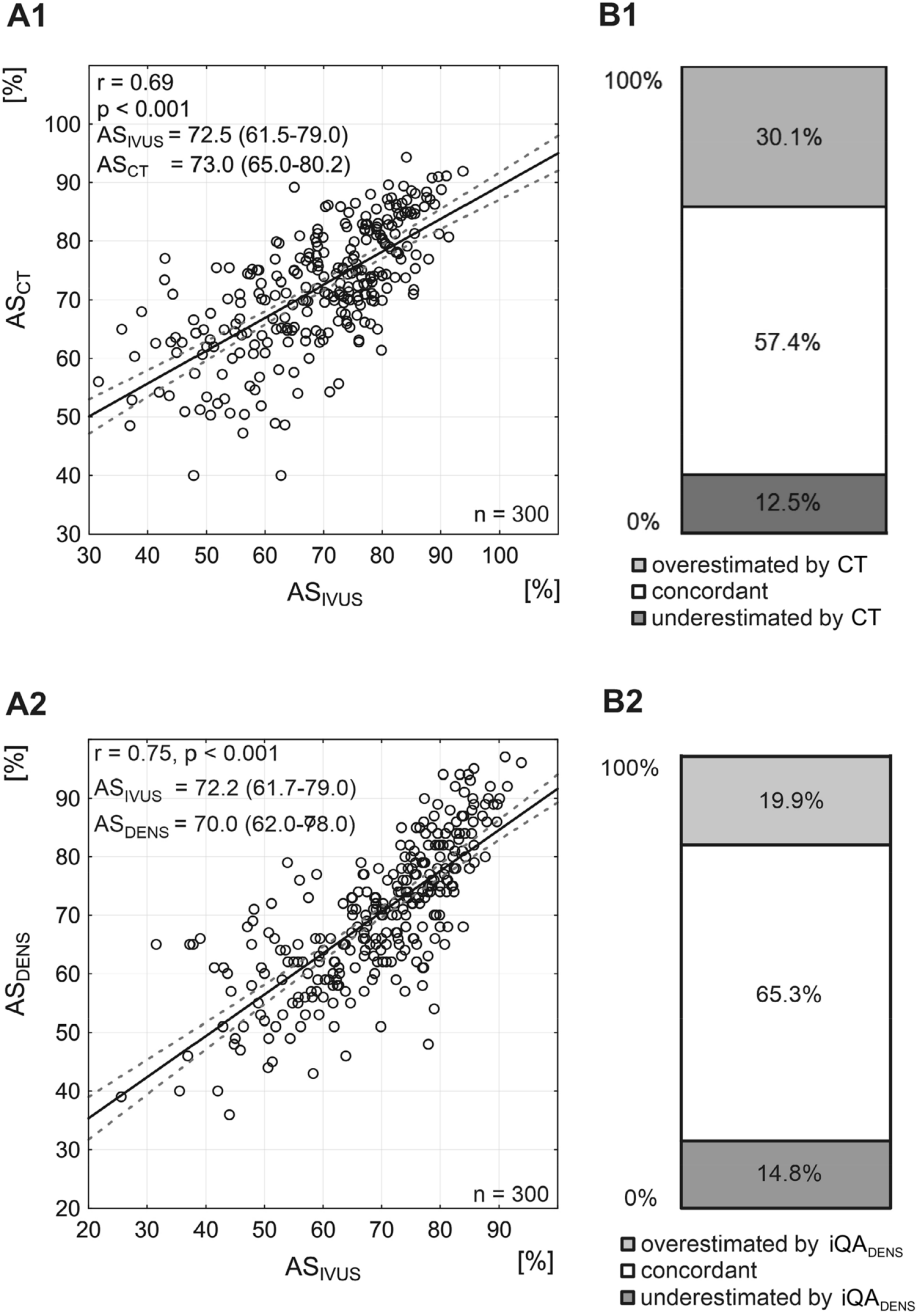
Relation between AS estimated by CTA/iQA_DENS_ and IVUS. The correlations (CTA—A1 and iQA_DENS_—A2) are moderate, linear and highly statistically significant. Distribution bars show proportions of CTA (B1) and iQA_DENS_ (B2) measurements concordant with IVUS (defined as falling within ± 10% of the IVUS-measured value) and the proportions of over- and underestimated measurements. Note that almost 60% of AS-CT measurements and more that 60% of AS-iQA_DENS_ are concordant with IVUS

**Fig. 4:**
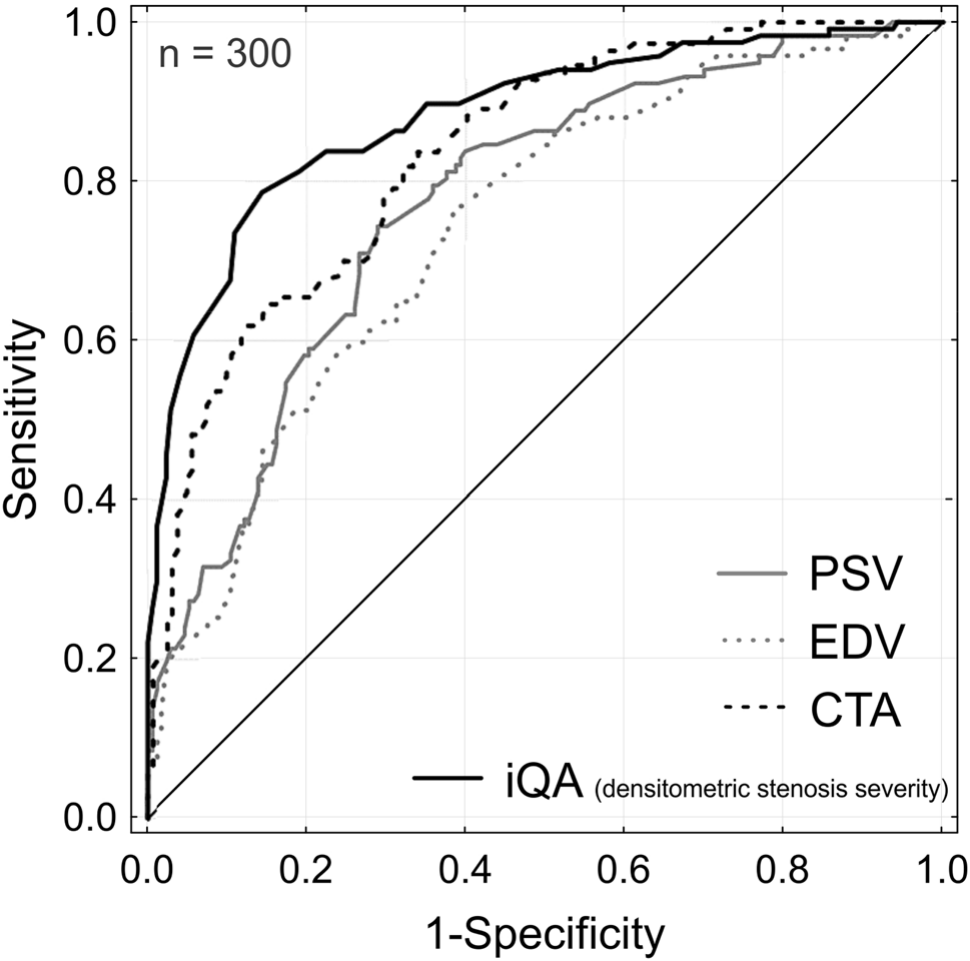
ROC predictors of ≥ 75% AS by IVUS. Receiver operating characteristic (ROC) curves showing overall accuracy of DUS, CTA and iQA in predicting ≥ 75% AS by IVUS. The highest accuracy was found for iQA. The overall accuracy was expressed by the area under the ROC curve (AUC) where 0.5 is no relationship and 1.0 is perfect relationship. For cutoff values, accuracy, positive and negative predictive values in predicting ≥ 50% and ≥ 75% AS by IVUS see Tables 2, 3, Suppl Fig. 4-I and Suppl Fig. 4-II

Figures 2–4 and Suppl Fig. 2 to Suppl Fig. 4 demonstrate the relationship between the routine imaging modalities and IVUS measurements.

There was a curvilinear relationship between DUS velocities and IVUS-AS (correlation coefficient of 0.51 and 0.45, respectively, *p* < 0.001 for both, Fig. 2) and, similarly, between DUS velocities (PSV, EDV) and IVUS-MLA (correlation coefficient of 0.49 and 0.42, respectively, *p* < 0.001 for both; Suppl Fig. 2-I).

Study data-derived mathematical formulas relating PSV and EDV with AS and MLA by IVUS according to the best-fit curves are provided in Fig. 2 and Suppl Fig. 2-I.

DUS MLD and RD measurements were performed in the first 100 (out of 126; 79.4%) study arteries with visualization considered sufficient to appropriately determine stenosis severity according to NASCET. There was only a moderate (though statistically significant) correlation between DUS-MLD and IVUS-MLD (r = 0.35, *p* < 0.001), DUS-RD and IVUS-RD (r = 0.33, *p* < 0.001), and DUS-DS and IVUS-DS (r = 0.41, *p* < 0.001). DUS systematically underestimated MLD and overestimated DS (Suppl Fig. 2-II, Suppl Fig. 2-III, and Suppl Fig. 2-IV, Suppl Table 1).

There was a good correlation between AS by CTA and IVUS (r = 0.69, *p* < 0.001, Fig. 3 and Suppl Fig. 3-I).

CTA measurements of AS were concordant with IVUS in 57.4% (CTA underestimation in 12.5% cases, overestimation in 30.1%; Fig. 3B1, Suppl Table 1). This occurred despite the CTA systematic underestimation of MLA (92.3% cases) and RA (80.4%) in relation to IVUS (Suppl Fig. 3-II D1 and Suppl Fig. 3-III D1, Suppl Table 1).

The largest proportion of measurements concordant with IVUS (65.3%, Fig. 3B2) occurred for densitometric evaluation of AS by iQA. With this technique, comparing automatically the density of the contrast column in the reference segment and at the point, it reaches a minimum, there was a similar proportion of under- and overestimated measurements (14.8% and 19.9% respectively, Fig. 3B2). The relationship between densitometric iQA and IVUS for MLA and RA measurements is presented in Suppl Fig. 3-II and Suppl Fig. 3-III. The densitometric iQA measurements highly correlated with IVUS (r = 0.75 for AS, r = 0.82 for MLA, r = 0.60 for RA, *p* < 0.001 for all). iQA measurements of DS showed concordance with IVUS-DS only in 41.1%. iQA underestimated the IVUS measurement in 7.9%, whereas overestimation in relation to IVUS occurred in 51.0% of cases (Suppl Fig. 3-IV D).

Overall, the correlation between DS by iQA and IVUS was good and statistically significant (r = 0.63, *p* < 0.001). Data for MLD and RD by iQA vs IVUS are given in Suppl Fig. 3-V and Suppl Fig. 3-VI respectively.

ROC analysis identified iQA densitometric measurement of the stenosis severity as the best predictor of IVUS-determined AS ≥ 75% (AUC 0.88, cutoff 74%, Fig. 4). Individual ROC analyses of the predictive values of DUS flow velocities and CTA for IVUS-DS ≥ 50% and IVUS-AS ≥ 75% are provided in Suppl Fig. 4-I and Suppl Fig. 4-II.

**Table 3:** IVUS validation of DUS flow velocities (PSV, EDV) and CTA area stenosis ≥ 75% against IVUS area stenosis ≥ 75%

AS_IVUS_ ≥ 75%							
	Cutoff	AUC	Sensitivity	Specificity	PPV	NPV	*p* value
PSV	2.58 m/s	0.77	0.74	0.71	0.64	0.80	< 0.001
EDV	0.75 m/s	0.74	0.77	0.61	0.57	0.80	< 0.001
CTA	72.4%	0.79	0.74	0.71	0.61	0.85	< 0.001
PSV&EDV	n/a	0.77	0.74	0.71	0.64	0.80	< 0.001 for PSV
							0.98 for EDV
PSV&EDV &CTA	n/a	0.85	0.79	0.82	0.74	0.85	0.32 for PSV
							0.34 for EDV
							< 0.001 for CT

On univariate model, PSV (ROC area under the curve, AUC 0.77, cutoff 2.6 m/s), EDV (AUC 0.72, cutoff 0.71 m/s) and CTA DS (AUC 0.83, cutoff 59.6%) were predictors of ≥ 50% DS by IVUS (*p* < 0.001 for all). Detailed data including sensitivity, specificity and the positive and negative predictive value of PSV, EDV and CTA in determining AS ≥ 75% and DS ≥ 50% by IVUS are given in Tables 3 and 4.

**Table 4:** IVUS validation of DUS flow velocities (PSV, EDV) and CTA diameter stenosis ≥ 50% against IVUS diameter stenosis ≥ 50%

DS_IVUS_ ≥ 50%							
	Cutoff	AUC	Sensitivity	Specificity	PPV	NPV	*p* value
PSV	2.6 m/s	0.77	0.62	0.83	0.87	0.52	< 0.001
EDV	0.71 m/s	0.72	0.68	0.66	0.80	0.51	< 0.001
CTA	59.6%	0.83	0.83	0.68	0.83	0.68	< 0.001
PSV&EDV	n/a	0.77	0.67	0.77	0.85	0.63	< 0.001 for PSV
							0.67 for EDV
PSV&EDV &CTA	n/a	0.85	0.75	0.81	0.88	0.63	0.23 for PSV
							0.46 for EDV
							< 0.001 for CT

The multivariable model eliminated PSV and EDV, leaving CTA as a sole (amongst those evaluated in the study) independent non-invasive diagnostic modality to determine carotid stenosis severity (*p* = 0.008).

## Discussion

Despite accumulating evidence for the role of carotid plaque morphology in relation to the risk of carotid-related cerebral injury and stroke [1–10], current guidelines continue to recommend using stenosis severity thresholds (usually 50% and 70% in symptomatic patients and 60% and 80% in asymptomatic subjects) as the principal parameter in clinical decision-making that includes revascularization by surgical or endovascular route [2, 3, 11–13].

This is the first prospective study comparing typically used non-invasive modalities (DUS and CTA) against the vascular imaging “gold standard” tool, IVUS, in evaluating the degree of carotid artery stenosis. DUS velocities (PSV, EDV) showed a highly significant though only moderate correlation (coefficient value of ≈0.4–0.5) with IVUS measurements of minimal lumen diameter and area stenosis. The relationship was yet weaker between the DUS NASCET and IVUS measurement of MLD and DS, confirming that DUS should not be used as a sole imaging modality to determine management in individuals with carotid stenosis [45–47]. Even with accurate lumen-artery border detection, the one-dimensional ultrasound imaging may lead to underestimation of MLD in case of irregular (eg., elliptic on cross-section) lesion. In our examination, only RD measurement reached better (moderate) accuracy – most likely due to regular, circular shape and better distance/spatial resolution ratio that are naturally more problematic for the minimal lumen.

Reduction of diameter stenosis (DS) on intra-arterial (catheter) quantitative angiography (iQA) has been the reference standard in reporting carotid artery stenosis severity as “% stenosis” [14, 26]. The iQA-DS measurement has been applied in pivotal revascularization trials as a sole [14, 26] or prevailing [48, 49] technique. We found that iQA evaluation of diameter stenosis is broadly consistent with IVUS; however, iQA tends to systematically overestimate DS against IVUS (Suppl Fig. 3-IV).

Our work shows that the sensitivity and specificity of PSV appear acceptable to detect IVUS-confirmed area stenosis of ≥ 75% with the PSV cutoff value of ≈2.6 m/s (Suppl Fig. 4-II), and similar findings were obtained for ≥ 50% DS (Suppl Fig. 4-I). However, for several reasons, DUS may fail in any precise determination of AS (and DS), particularly in less severe lesions [22, 48, 49]. First, for anatomic reasons DUS may not show the site of MLD in non-concentric lesions in particular. Another important factor that may reduce the overall accuracy of DUS with respect to the flow velocities may be the presence of contralateral carotid occlusion, leading to the unilateral DUS velocities rise associated with compensatory blood flow increase [52, 53]. It has been suggested that the accuracy of non-invasive imaging for the evaluation of cervical carotid artery stenosis may be generally overestimated in the literature [54–56]. This is relevant to clinical practice as—following the methodology of some large trials [23, 25]—up to 40% CEAs may still be performed today based on isolated DUS measurements [57]. Series comparing non-invasive methods with iQA indicate that the grading of carotid stenosis as medical or potentially surgical remains uncertain in a relatively high proportion of patients, suggesting the use of two imaging modalities for decision-making on revascularization [2]. With DUS use for stenosis degree evaluation, 1 out of 6 arteries would be reclassified by CTA [58]. It should be emphasized that DUS alone is not sufficient to distinguish a non-significant vs borderline (such as 50–60%) carotid stenosis with adequate accuracy [59, 60]. This observation is of crucial importance as border-line stenosis (50% for symptomatic and 60% for asymptomatic patients) is a threshold for intervention according to current guidelines [2, 3, 11–13]. Recent meta-analysis including 809 carotids confirmed a very poor sensitivity (31%) while it indicated a sufficient specificity (84%) for grading 50–69% stenosis with DUS as compared to iQA. DUS accuracy for grading 70–99% carotid stenosis seems to have a higher sensitivity (83%) but much lower specificity (54%) [54]. However, higher rates of DUS-related misclassifications have been reported, particularly in large-diameter arteries [61]. A recent study showed that the qualification to surgical treatment of carotid artery stenosis suffers from a wide variability in carotid velocity thresholds in different DUS laboratories [62]. This directly influences the treatment decision-making [60].

Even though PSV and EDV were univariate predictors of ≥ 75% IVUS-AS (and ≥ 50% IVUS-DS) in the present study (Suppl Fig. 4-II, Suppl Fig. 4-I), they were both eliminated by CTA in multivariate analysis (Tables 3, 4). Although this might suggest using CTA as a single diagnostic tool, DUS remains the primary screening modality due to a non-negligible risk of CTA contrast-related complications and cost [2, 3, 11–13]. We found that although CTA systematically underestimates both MLA and RA, it is highly accurate in AS evaluation (Fig. 3 A1 and B1, Suppl Fig. 3-II A1-D1, Suppl Fig. 3-III A1-D1). CTA does not differentiate between the systolic and diastolic flow whereas IVUS takes (in particular—reference) measurements when the vessel reaches its greatest diameter that may be particularly relevant for the reference segment measurements [63]. Moreover, CT diagnostic accuracy is markedly reduced with lesion calcification [64, 65]. Despite these limitations, not only CTA accuracy in detecting ≥ 50% DS and ≥ 75% AS is greater than that of DUS, but also—according to the present analysis – the overall diagnostic accuracy is not increased significantly by adding DUS in the ROC analysis (Tables 3, 4).

An optimal carotid stenosis screening tool should be both highly sensitive and highly specific (Fig. 4, Suppl Fig. 4-I, Suppl Fig. 4-II). When considering DUS as a screening tool, the velocity threshold should optimally be decreased to the value joining high sensitivity with reasonable loss in specificity to reduce the proportion volume of false-negative results and reduce, at the same time, the number of other examinations (such as CTA) needed for a cross-verification. With the use of only one diagnostic non-invasive method (DUS in particular), a significant proportion of patients may be misclassified into a discordant category (surgical vs. medical), providing an argument for performing both types of non-invasive imaging [58]. In case of discordance between the non-invasive methods, intra-arterial angiography may be needed to determine lesion severity [20, 21, 42, 43]. Once iQA is performed, IVUS may accurately visualize the MLA and RA, assess plaque morphology [39], and provide procedural quality control [66–68].

Important novel information from this study is the high diagnostic accuracy of iQA densitometric measurement (density of a contrast column in MLD in a relation to a RD) that was found to be greater than that of the conventionally-used iQA diameter stenosis.

The magnitude of imaging modality-dependent variations in the numeric “value” of stenosis severity identified in the present study suggests that evidence-based decision-making should consult individual clinical trials and studies with respect to the specific modality used to determine stenosis severity. In case of discrepancies, at least CTA should be employed.

Carotid stenosis severity remains important clinically, as totality of current evidence suggests that patients with increased-stroke-risk asymptomatic carotid stenosis of “60–99%” should be considered for low-risk carotid revascularization on top of maximized medical therapy that is now known to be unable to sufficiently control stroke risk in relation to carotid stenosis [69].

### Limitations

Our work has evaluated two non-invasive methods, DUS and CTA, in relation to IVUS. We have not been able to include MRA in our analysis because this technique is rarely used in our institution and in referring hospitals. This is consistent with overall rather limited use of MRA in diagnosing carotid stenosis and determining its severity in clinical practice of carotid revascularization [70]. In a recent analysis of imaging prior to CEA in nearly 20,000 procedures in the Vascular Quality Initiative database, MRA was the sole imaging modality in only 2.0% patients whereas DUS and MRA were performed in 9.4% [70].

Other potentially significant shortcomings of our work, in the context of DUS validation with IVUS, may arise from our analysis limited to the cross-sectional stenosis severity. Other factors, such as cardiac output and arterial blood pressure/peripheral vascular resistance [53], contralateral carotid artery occlusion (or severe stenosis) and lesion length may affect DUS velocities. Our pilot analysis indicated that automated pullback of the IVUS catheter tends to be uneven at carotid bifurcations, precluding measurements of the lesion length [71]; a problem similar to the effect of movement artifacts raised previously by other investigators in coronary bifurcations [72]. In addition, in the carotids, there is also an additional, prominent, “jumping” of the IVUS probe, back-and-forth, with the heartbeat [71]. The role of contralateral carotid occlusion (if present) and the potential role of the lesion length on DUS flow velocities require further evaluation. Furthermore, the CCA/ICA PSV ratio was not included into analysis as a result of lack of routine recordings of the CCA velocities. Finally, our analysis intentionally excluded patients with highly calcified lesions as for those patients it is not possible to determine precisely stenosis severity using DUS and CTA [73, 74].

### Clinical implications

Stenosis severity directly affects treatment decisions in patients with both symptomatic or asymptomatic carotid stenosis. Our work identified significant imaging modality-dependent variations in carotid stenosis severity determination using IVUS validation. Analysis of the value of routine imaging modalities such as DUS and CTA against imaging gold standard, IVUS, confirmed that Doppler ultrasound flow velocities are not a reliable predictor of stenosis severity especially in ‘borderline’ stenosis. Furthermore, we found that CTA overestimated stenosis severity in more than 30% of patients. This means that some patients operated solely on CTA imaging might not benefit from this treatment. This finding is consistent with recent data by Horev et al. [73] who compared measurements of carotid stenosis severity using iQA and CTA. These investigators demonstrated that out of 90 patients with significant stenosis on CTA (thus being candidates for CEA), only 70 had a significant stenosis on iQA. Thus, the CTA overestimation error of “% diameter stenosis” wrongly classified 22% of lesions (patients) to the revascularization cohort; a finding consistent with our results (Fig. 3). Our findings support the notion of Horev et al. [73] that despite ongoing radiological progress, the specificity of CTA in accurately assessing carotid stenosis remains relatively low; consequently, patients could be referred for unnecessary CEA surgery and may become exposed to associated potential complications [73].

Invasive angiography correlated best with IVUS imaging. This is important because invasive angiography has been the pivotal imaging technique in initial trials of carotid revascularization in primary and secondary stroke prevention that provided basis for clinical guidelines [14, 15] In more recent quality trials such as CREST-1 [26] or ACT-1 [24] intraarterial angiography has been employed as a prevailing technique in case of discrepancies in non-invasive imaging. IVUS is an invasive technique, and it would be impractical and costly to routinely perform IVUS in patients referred for carotid revascularization. IVUS, as the ‘final’ verification technique will be reserved for patients in whom invasive angiography is ambiguous.

## Conclusions

Physicians should be aware of the variability in carotid stenosis severity determined using different imaging modalities. The stenosis severity evaluation method(s) should be taken into consideration when applying clinical trial data as the basis for clinical decision-making.

### Supplementary Information

Below is the link to the electronic supplementary material.

Supplementary file1 (DOCX 2270 KB)
