# Supplementary material for: Imaging modality-dependent carotid stenosis severity variations against intravascular ultrasound as a reference: Carotid Artery intravasculaR Ultrasound Study (CARUS)
Source: Int J Cardiovasc Imaging. 2023 Aug 21;39(10):1909–20. doi: 10.1007/s10554-023-02875-1 (PMC10589130; doi:10.1007/s10554-023-02875-1)
Supplement: Supplementary file 1 — Supplementary file1 (DOCX 2270 KB) [file 10554_2023_2875_MOESM1_ESM.docx]

**Supplementary data**

**Suppl Fig 1. Virtual histology-based plaque composition showed in Fig 1.**

**
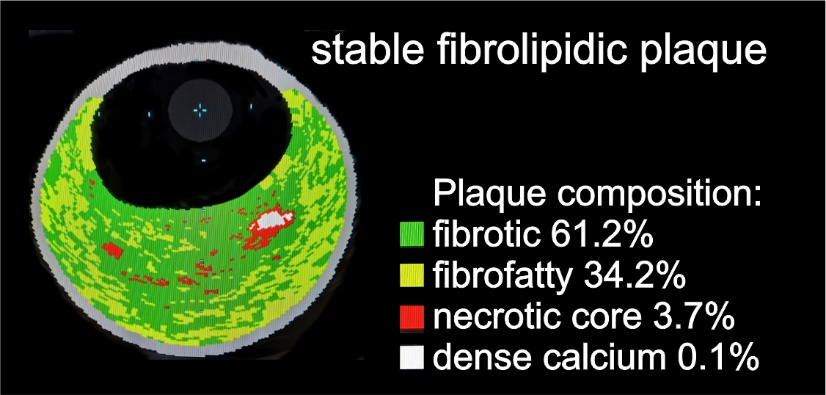
**

Virtual histology shows stable fibrolipidic plaque[38,39] and absence of increased-stroke-risk characteristics such as a thin fibrous cap and a large confluent necrotic core content.

**Suppl Fig 2-I. Relation between DUS flow velocities (PSV, EDV) and minimal lumen area measured by IVUS.**

**
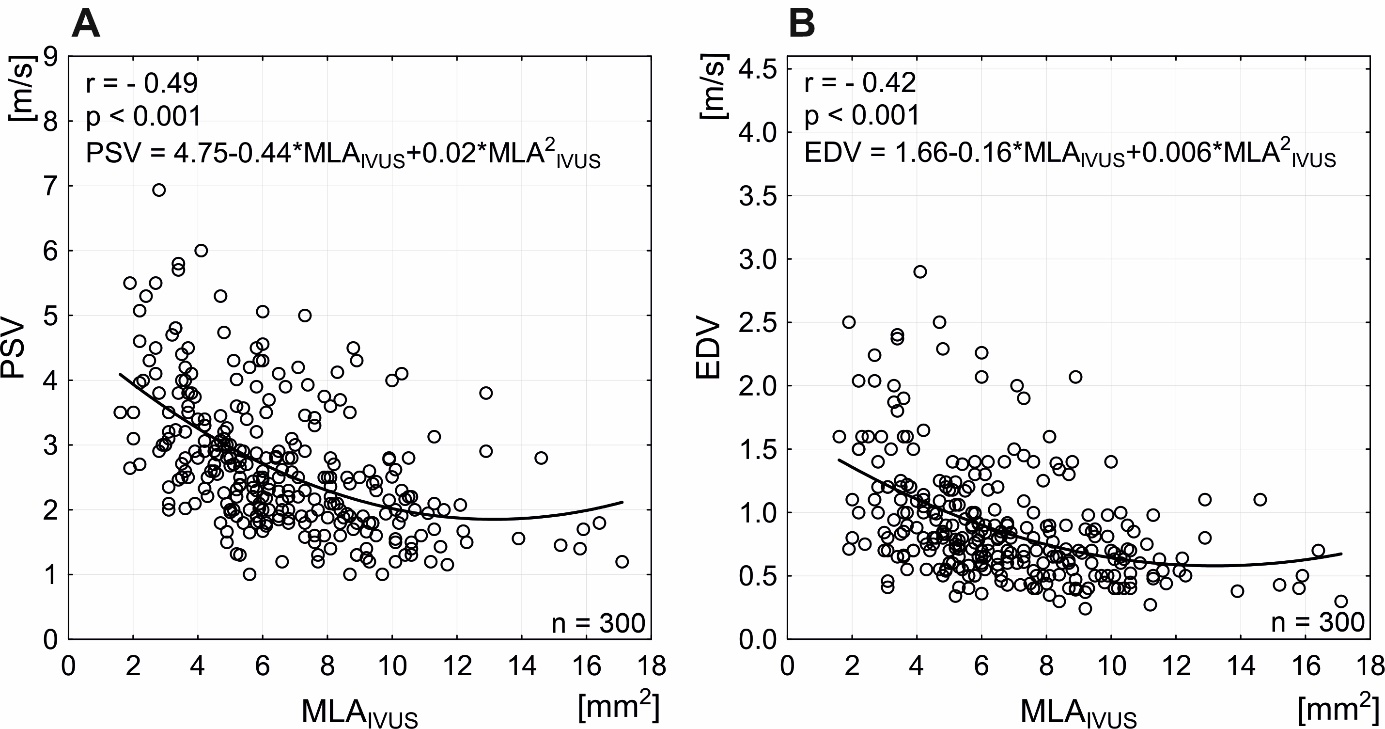
**

Note that overall correlations here are moderate, curvilinear and highly statistically significant for both comparisons (correlation coefficient “r” 0 indicates absence of any correlation whereas 1 indicates a perfect correlation). Best-fit mathematical formulas are provided for both relationships.

**Suppl Fig 2-II.** Relation between DS estimated by DUS and IVUS.


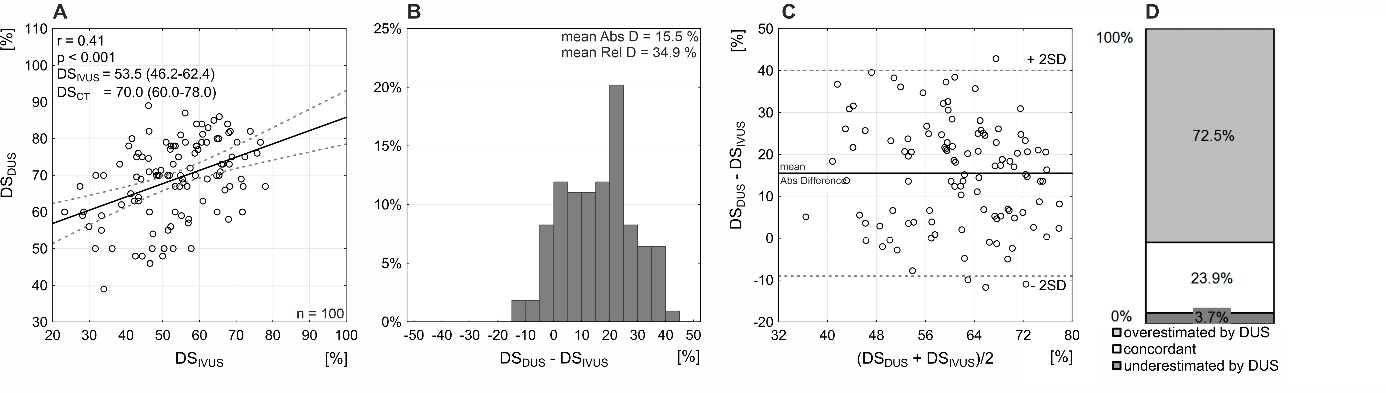
The correlation here is linear but weak though highly statistically significant (A). Bar chart showing absolute and relative difference between DS estimated by DUS and IVUS (B). Bland-Altman plot showing agreement degree for DS between DUS and IVUS (C). Distribution bar (D) shows the proportion of DS measurements concordant and those over-/underestimated by DUS against IVUS (D). Note that more than 70% of DUS results are overestimated, i.e. DUS indicates DS values significantly greater than determined with IVUS.

**Suppl Fig 2-III. Relation between MLD estimated by DUS and IVUS.**


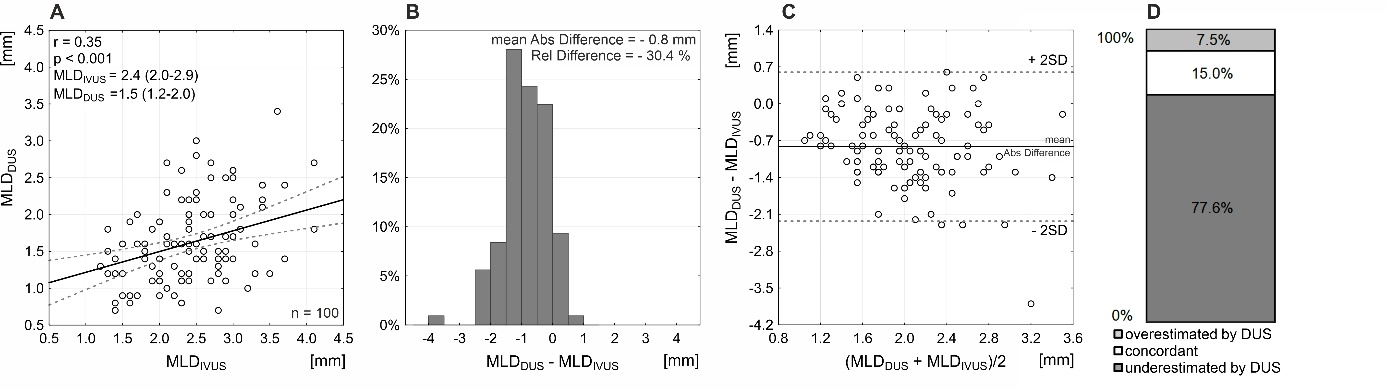
The correlation is weak, linear and highly statistically significant (A). Bar chart showing the absolute and relative difference between MLD estimated by DUS and IVUS (B). Bland-Altman plot showing agreement degree for MLD between DUS and IVUS (C). Distribution bar (D) shows rate of concordant measurements and those over-/underestimated for MLD by DUS against IVUS (D). Note that ≈80% DUS measurements are underestimated, i.e. DUS indicates MLD values significantly smaller than the IVUS values.

**Suppl Fig 2-IV.** Relation between RD estimated by DUS and IVUS.


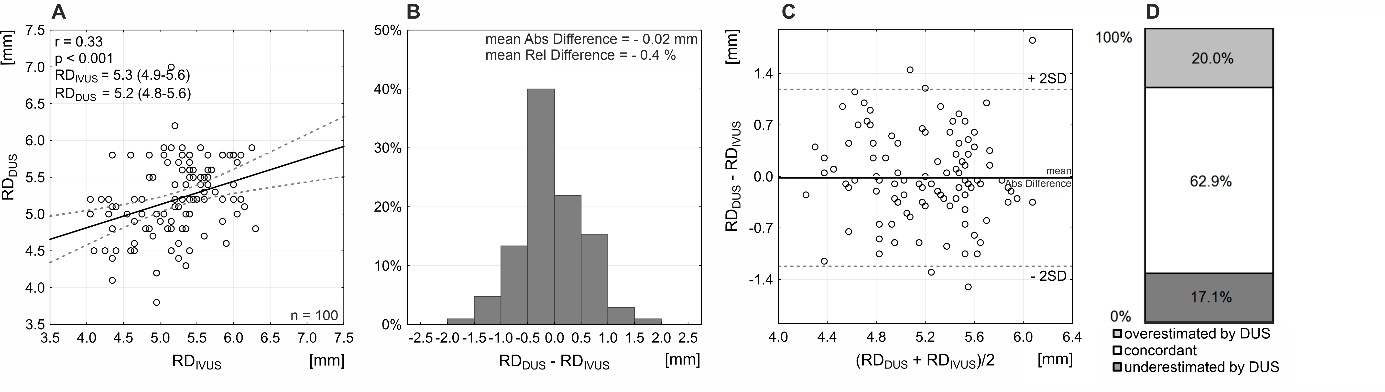
The correlation is weak, linear and highly statistically significant (A). Bar chart showing absolute and relative difference between RD estimated by DUS and IVUS (B). Bland-Altman plot showing agreement degree for RD between DUS and IVUS (C). Distribution bar (D) shows the proportion of measurements concordant and those over-/underestimated for RD by DUS against IVUS. Note that ≈60% DUS results are concordant with IVUS, suggesting that DUS may be a reasonable method to evaluate RD.

**Suppl Fig 3-I.** Relation between AS estimated by CTA/iQA_DENS_ and IVUS (cont).


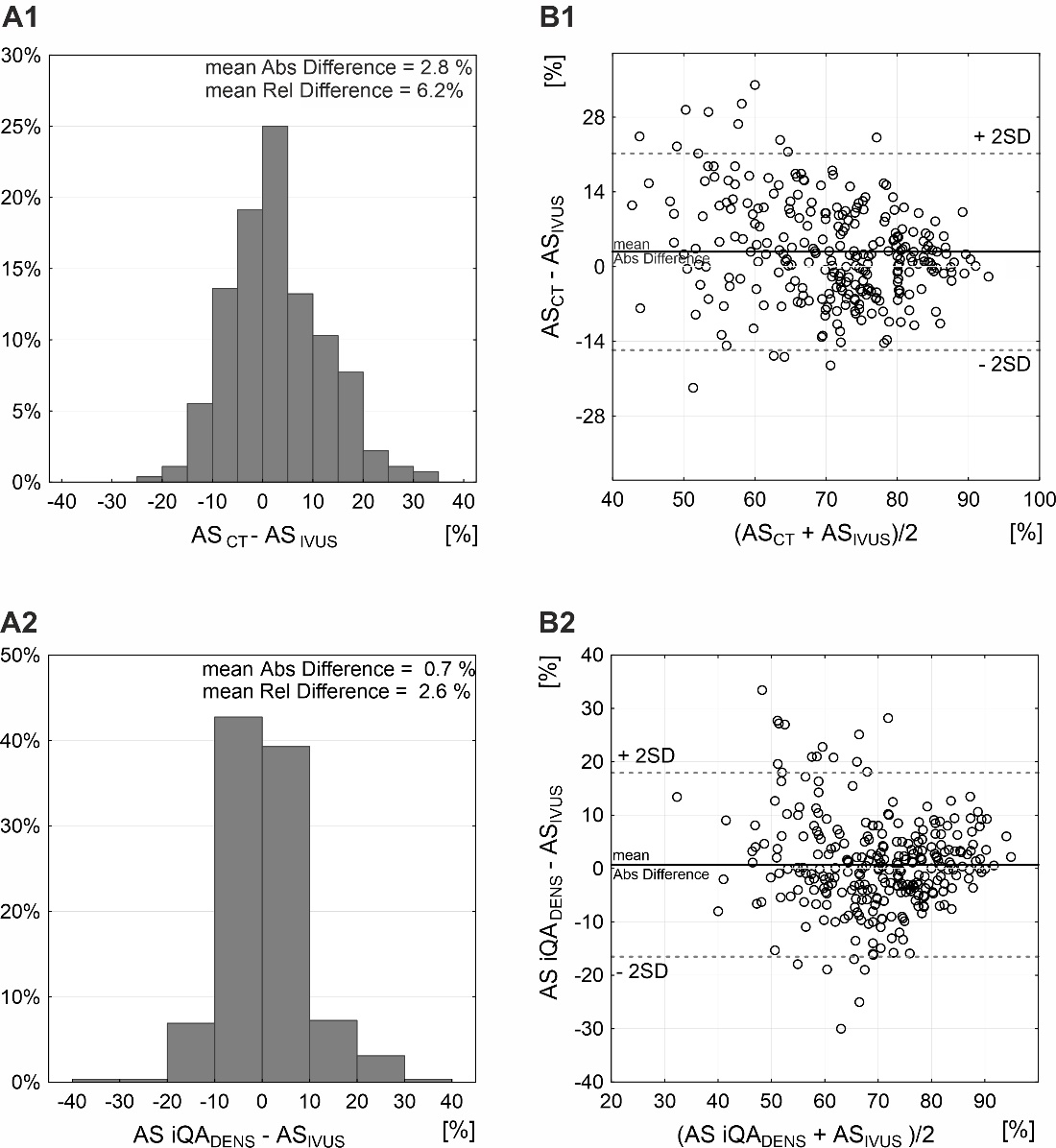


Histograms show absolute and relative difference between AS-CT (A1), AS-iQA_DENS_ (A2) and IVUS. Bland-Altman plots shows a good agreement between CTA (B1), iQA_DENS_ (B2) and IVUS for AS evaluation.

**Suppl Fig 3-II.** Relation between MLA estimated by CT/iQA_DENS_ and IVUS.


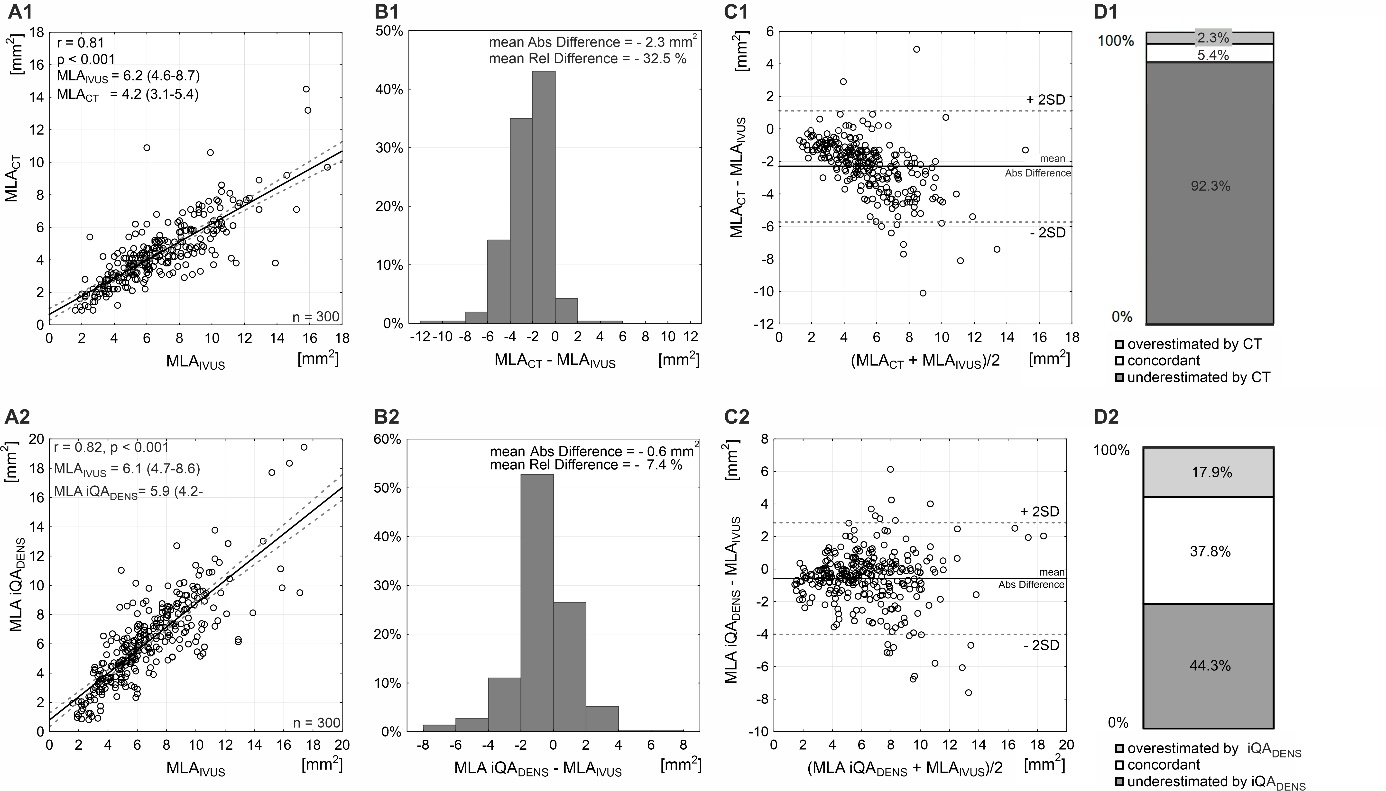


For CT derived MLA (upper row), the correlation is strong, linear and highly statistically significant (A1). Bar chart showing absolute and relative difference between MLA estimated by CT and IVUS (B1). Bland-Altman plot showing agreement degree for MLA between CT and IVUS (C1). Distribution bar (D1) shows proportion of concordance and over- and underestimation for MLA by CT against IVUS. Note that more than 90% of CT results are underestimated, i.e. CT indicates MLA values significantly smaller than IVUS values.

For densitometric iQA derived MLA (bottom row), the correlation is strong, linear and highly statistically significant (A2). Bar chart showing absolute and relative difference between MLA estimated by densitometric iQA and IVUS (B2). Bland-Altman plot showing agreement degree for MLA between densitometric iQA and IVUS (C2). Distribution bar (D2) shows the proportion of MLA measurements concordant and those over-/underestimated by densitometric iQA against IVUS. Note that 44.3% densitometric iQA results are underestimated against IVUS, i.e. significant proportion of densitometric iQA indicates MLA values significantly smaller than IVUS values.

**Suppl Fig 3-III.** Relation between RA estimated by CT/iQA_DENS_ and IVUS.


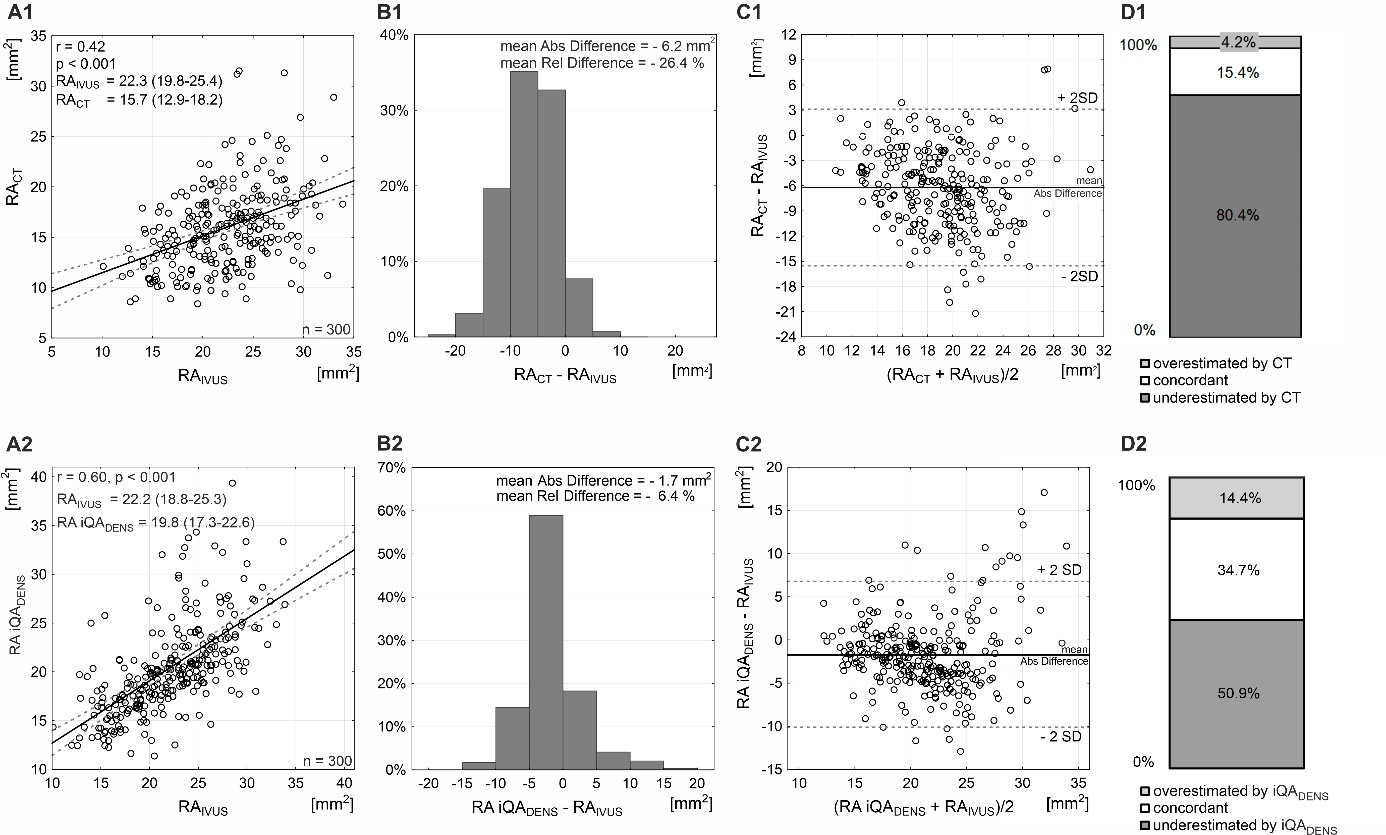


For CT derived RA, the correlation is weak, linear and highly statistically significant (A1). Bar chart showing absolute and relative difference between RA estimated by CT and IVUS (B1). Bland-Altman plot showing agreement degree for RA between CT and IVUS (C1). Distribution bar (D1) shows the proportion of RA measurements concordant and those over-/underestimated against IVUS. Note that ≈80% CT results are underestimated, i.e. CT indicates RA values significantly smaller than IVUS values.

For densitometric iQA derived RA, the correlation is strong, linear and highly statistically significant (A2). Bar chart showing absolute and relative difference between RA estimated by densitometric iQA and IVUS (B2). Bland-Altman plot showing agreement degree for RA between densitometric iQA and IVUS (C2). Distribution bar (D2) shows the proportion of RA measurements concordant and those over-/underestimated by densitometric iQA against IVUS. Note that 50.9% densitometric iQA results are underestimated, i.e. significant proportion of densitometric iQA readings indicates RA values significantly smaller than IVUS values.

**Suppl Fig 3-IV Relation between DS estimated by iQA and IVUS.**


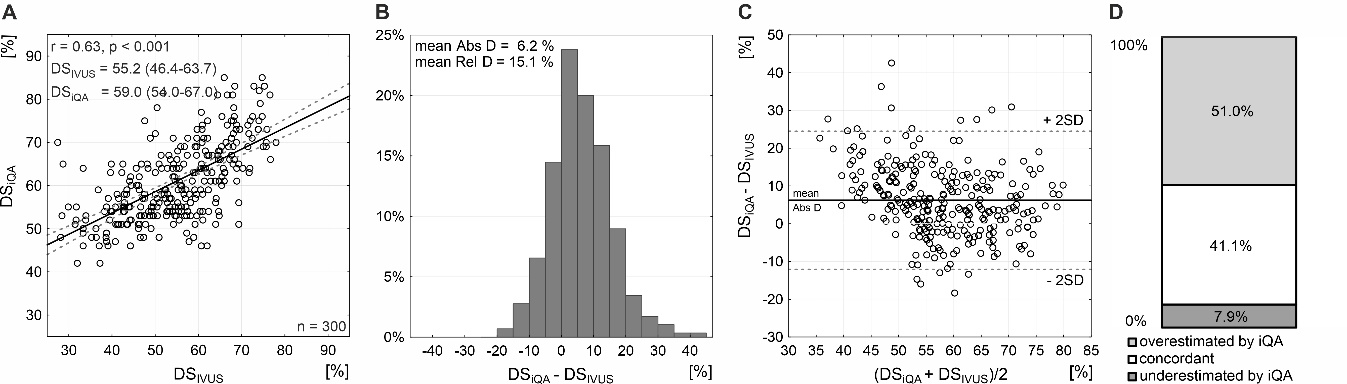
The correlation is linear and highly statistically significant (A). Bar chart showing absolute and relative difference between DS estimated by iQA and IVUS (B). Bland-Altman plot showing good agreement degree for DS between iQA and IVUS (C). Distribution bar (D) shows rate of concordance and over-/ underestimation for iQA DS against IVUS DS. Note that 41.1% iQA measurements are concordant with IVUS, whereas majority are overestimated (ie, iQA shows a greater degree of stenosis than IVUS used as reference).

**Suppl Fig 3-V.** Relation between MLD estimated by iQA and IVUS.


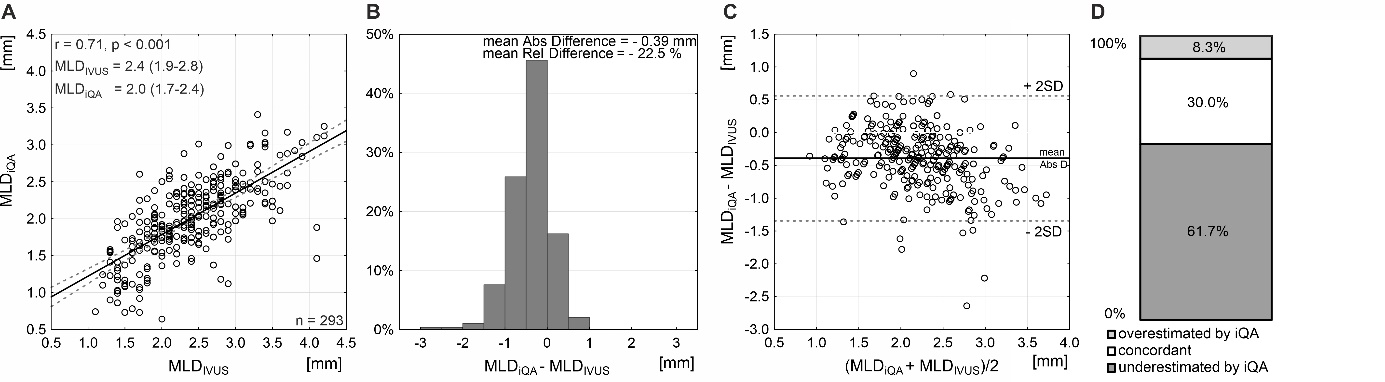
The correlation is strong, linear and highly statistically significant (A). Bar chart showing absolute and relative difference between MLD estimated by iQA and IVUS (B). Bland-Altman plot showing agreement degree for MLD between iQA and IVUS (C). Distribution bar (D) shows the proportion of MLD-iQA measurements concordant and those over-/underestimated against IVUS (D). Note that ≈60% iQA results are underestimated, i.e. iQA indicates MLD values significantly smaller than IVUS values.

**Suppl Fig 3-VI.** Relation between RD estimated by iQA and IVUS.


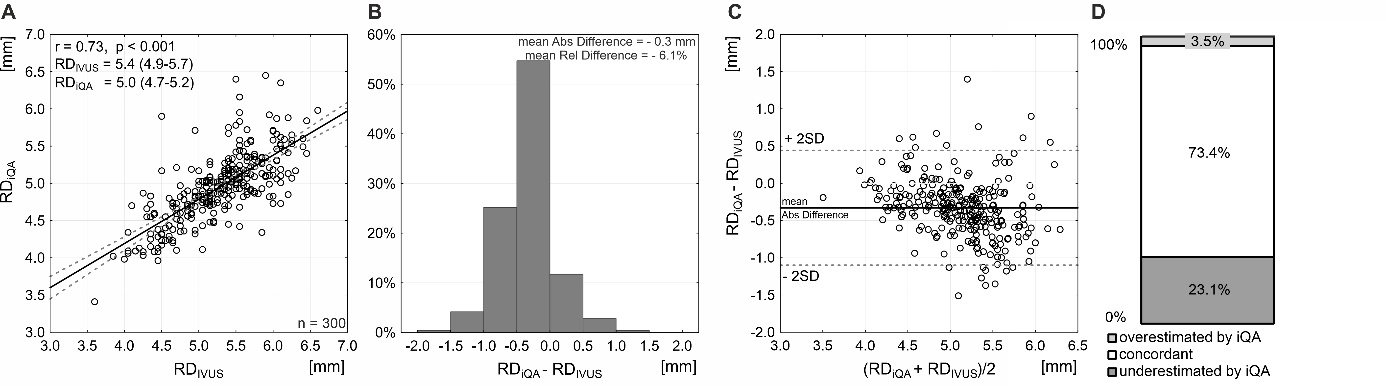


The correlation is strong, linear and highly statistically significant (A). Bar chart showing absolute and relative difference between RD estimated by iQA and IVUS (B). Bland-Altman plot showing agreement degree for RD between iQA and IVUS (C). Distribution bar (D) shows the proportion of RD-iQA measurements concordant and those over-/underestimated against IVUS (D). Note that more than 70% of iQA results are concordant with IVUS; one of the highest concordance proportions in this study.

**Suppl Fig 4-I. ROC predictors of ≥50% DS by IVUS.**


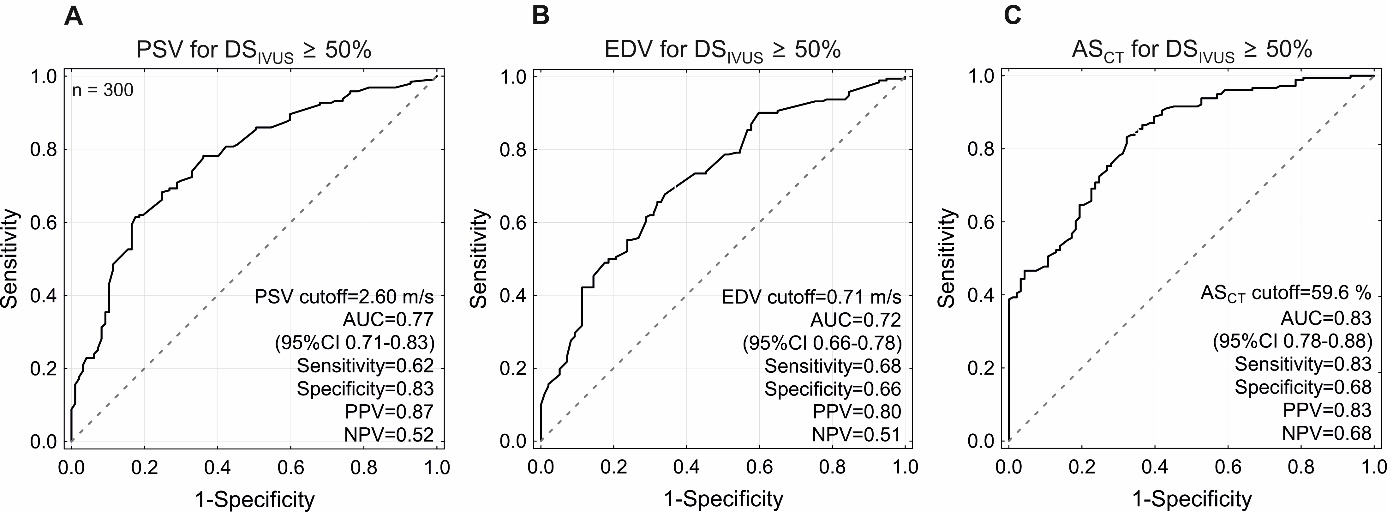


Receiver operating characteristic (ROC) curves showing overall accuracy of DUS PSV (A), DUS EDV (B) and AS CTA (C) for prediction of 50% DS by IVUS. The overall accuracy was expressed by the area under the ROC curve (AUC) where 0.5 is no relationship and 1.0 is perfect relationship.

**Suppl Fig 4-II. ROC predictors of ≥75% AS by IVUS.**


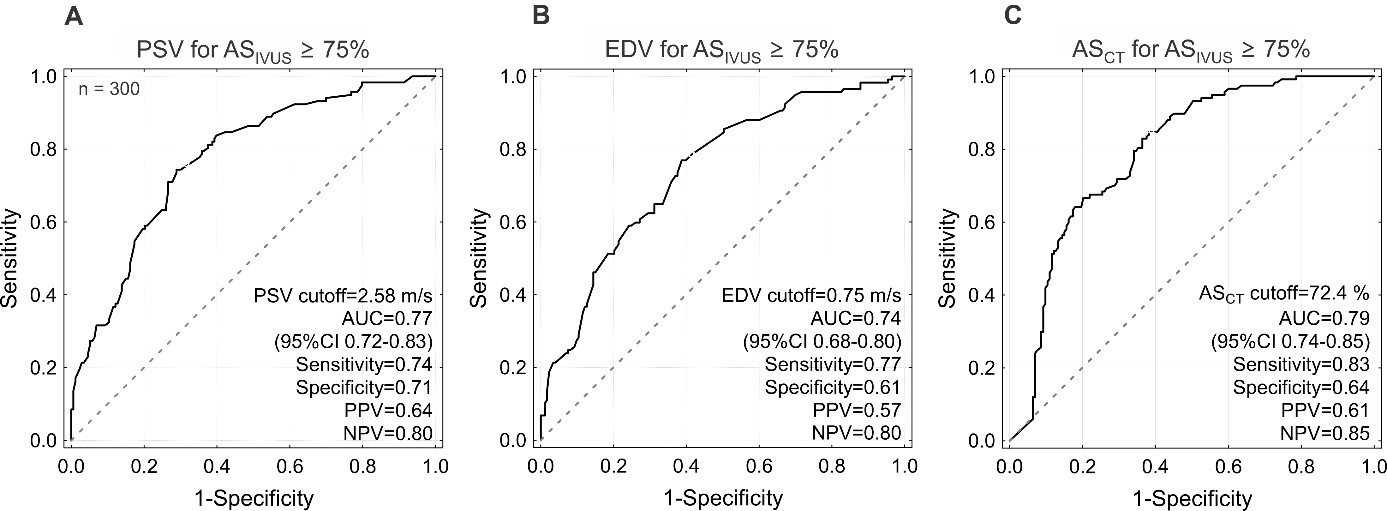


Receiver operating characteristic (ROC) curves showing overall accuracy of DUS PSV (A), DUS EDV (B) and AS CTA (C) for prediction of 75% AS by IVUS. The overall accuracy was expressed by the area under the ROC curve (AUC) where 0.5 is no relationship and 1.0 is perfect relationship.

**Suppl Table 1. Mean absolute difference, degree of proportion of measurements concordant and over/underestimated by DUS and CTA against IVUS**

|  | mean absolute difference (Bland-Altman) | measurements concordant* with IVUS  (% cases) | underestimated against IVUS (% cases) | overestimated against IVUS (% cases) | | | p-value for proportion of under- vs. overestimated |
| --- | --- | --- | --- | --- | --- | --- | --- |
| **DUS** | | | | | | | |
| MLD | -0.81 mm | 15.0 | 77.6 | | 7.4 | <0.001 | |
| RD | -0.02 mm | 62.9 | 17.1 | | 20.0 | 0.65 | |
| DS | +15.51% | 23.9 | 3.6 | | 72.5 | <0.001 | |
| **CTA** | | | | | | | |
| MLA | -2.3mm^2^ | 5.4 | 92.3 | | 2.3 | <0.001 | |
| RA | -6.2mm^2^ | 15.4 | 80.4 | | 4.2 | <0.001 | |
| AS | +2.79% | 57.4 | 12.4 | | 30.2 | <0.001 | |

*defined as within ±10% of the IVUS measurements value
